# Role of Point-of-Care Cerebral Ultrasonography in Determining the Etiology of Post-carotid Endarterectomy Deficit: A Case Report

**DOI:** 10.7759/cureus.64225

**Published:** 2024-07-10

**Authors:** Rida Touab, Abdeltif Chlouchi, Amine Meskine, Mohamed Drissi, Hicham Balkhi

**Affiliations:** 1 Anesthesia and Critical Care, Mohamed V Military Teaching Hospital, Rabat, MAR; 2 Cardiovascular Anesthesia and Critical Care, Mohamed V Military Teaching Hospital, Rabat, MAR

**Keywords:** reperfusion, ultrasound, intracerebral hemorrhage, hemiplegia, carotid endarterectomy

## Abstract

Carotid revascularization surgery is notorious for its neurological morbimortality. We report the case of a 74-year-old hypertensive patient, who underwent left internal carotid artery endarterectomy for a 90% stenosis under general anesthesia, presenting in the immediate postoperative period with right hemiplegia without consciousness disorders. Evaluation by cerebral ultrasound at bedside led to suspicion of intracerebral hemorrhage, which was confirmed by cerebral CT scan. The patient was treated by neuroresuscitation measures in the absence of the possibility of surgical intervention. This hemorrhage may be explained by a reperfusion injury due to the loss of cerebral autoregulation of these vessels, the loss of controlling blood pressure, and the use of heparin in vascular surgery. This is a rare but fatal complication with a high mortality rate.

## Introduction

Carotid revascularization surgery is notorious for its neurological morbimortality. The incidence of these complications is correlated with symptomatology, ranging from 2.4% to 5.5% [1], and can reach 20% in the acute phase. Although it is essentially ischemic, it opens the debate on the means of intraoperative neurological monitoring and anesthetic technique. Intracerebral hemorrhage after carotid surgery can occur in 0.7% of cases, usually within a few hours, and often has catastrophic consequences [2]. Several factors can raise the hemorrhagic risk such as anticoagulant use and blood pressure control failure [3]. CT scan remains the gold standard for diagnosis, but cerebral echo Doppler can identify the cause of neurological deterioration and its impact and assess the therapeutic measures.

## Case presentation

A 74-year-old gentleman, with chronic hypertension treated with dual therapy (angiotensin II receptor blocker (ARB II) and calcium channel blocker), presented with a left internal carotid artery stenosis discovered during a routine physical examination with imaging showing a 90% stenosis. He was scheduled for surgical endarterectomy under general anesthesia. The pre-anesthetic evaluation was favorable, and treatment management consisted of suspending ARB II for 24 hours before anesthesia and continuing the calcium channel blocker, including on the day of surgery, while following the preoperative fasting period.

**Figure 1:**
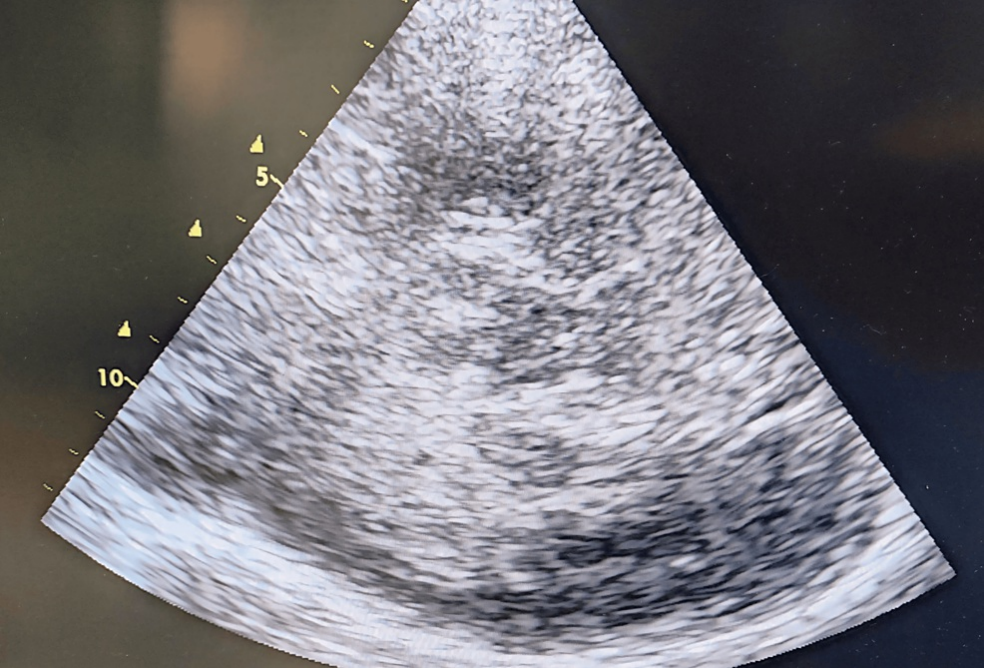
Hyperechoic image on cerebral ultrasound in relation to an intraparenchymal hematoma.

**Figure 2:**
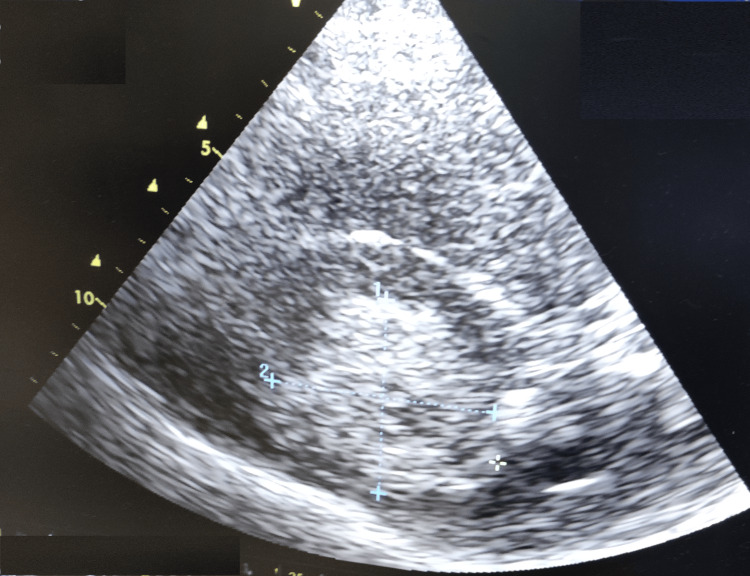
Hyperechoic image on cerebral ultrasound in relation to an intraparenchymal hematoma.

**Figure 3:**
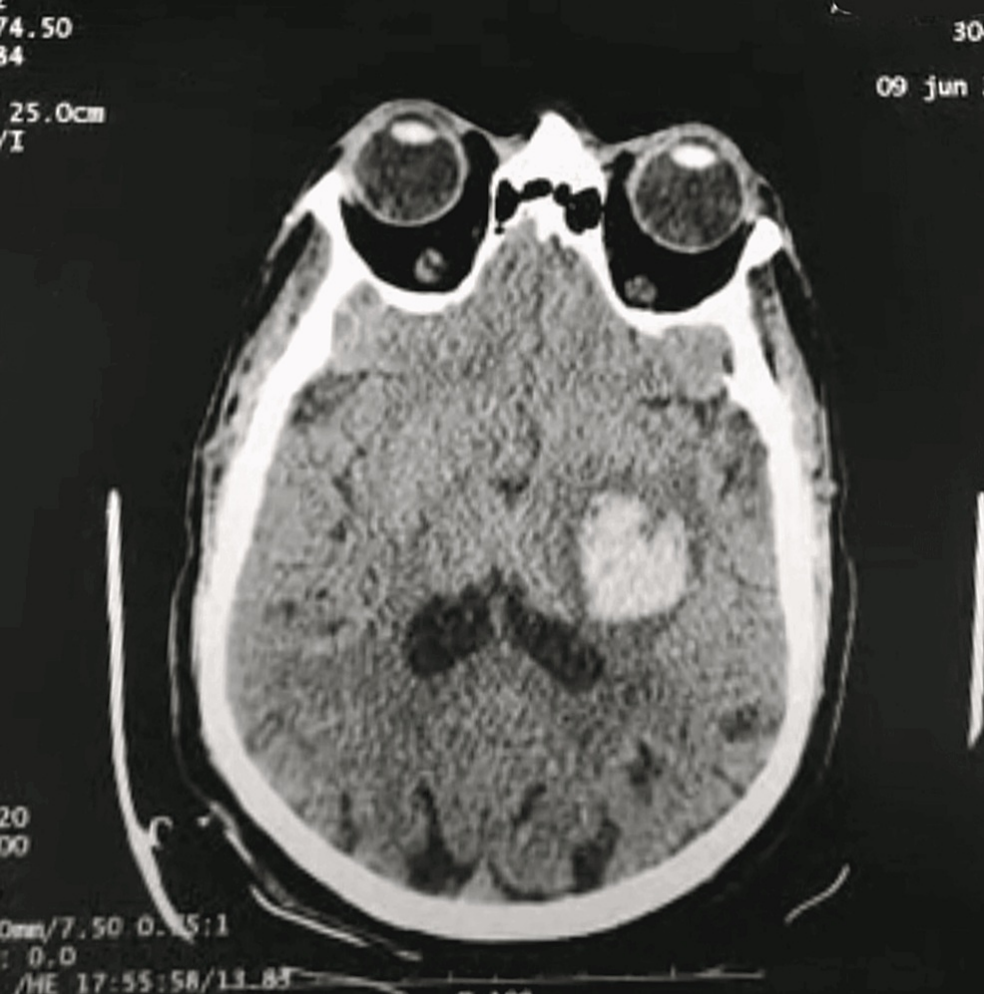
Caudate nucleus hematoma.

The one-hour surgical procedure was completed uneventfully under general anesthesia. This period included induction of anesthesia, intubation, and maintenance via inhaled halogenes. Intraoperative monitoring included a cardioscope, non-invasive blood pressure, pulse oximeter, and respiratory measurements (airway pressure, capnometry). He had no significant variations in blood pressure, rhythm, or oxygenation disorders. The systolic blood pressure was maintained between 120 and 150 mmHg. On awakening, the patient presented a flaccid right hemiplegia with right hemiasomatognosia, retaining contact and execution of simple commands on the left, with stable hemodynamic and respiratory status, and no hypertension. Given the deficit, the patient was kept on artificial ventilation, and MRI was discussed. While waiting, a cerebral ultrasound scan was performed, revealing a hyperechoic image (Figures 1, 2) with midline deviation, raising suspicion of intraparenchymal bleeding, leading to a cerebral CT scan confirming the diagnosis (Figure 3).

**Figure 4:**
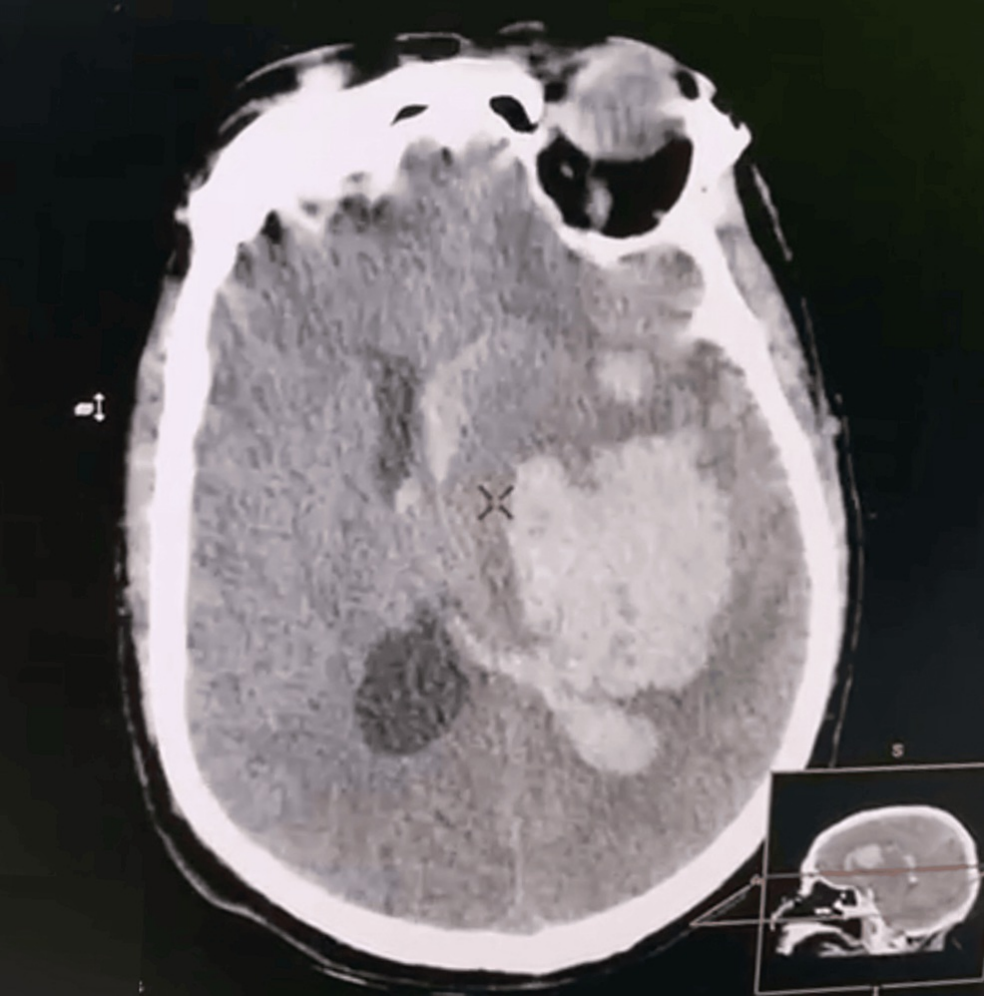
Worsening of intracerebral hemorrhage and mass effect.

The hematoma was deep, involving the temporoparietal internal capsule, and extending into the homolateral caudate nucleus, with intraventricular hemorrhage visible on the homolateral occipital horn, and not accessible to evacuation neurosurgery. The patient was kept ventilated under sedation and arterial pressure control, with transcranial Doppler and ACSOS monitoring. Rebleeding was suspected given diastolic villous degradation on DTC and confirmed on a repeat CT scan (Figure 4). A decompressive craniectomy was refused by the family and an unfavorable evolution was noted.

## Discussion

Our observation raises two points for discussion. First, the occurrence of a hemorrhagic complication in the immediate aftermath of a surgery reputed to have ischemic complications. Second, the value of bedside cerebral ultrasound for orientation of the morphological anomaly causing the neurological deficit.

The incidence of intracerebral hemorrhage following carotid stenting ranges from 0.36% to 6.67%, with a high mortality rate of 75% [4]. It occurs in 70% of cases during the first 24 hours. The pathophysiology of post-coronary revascularization hemorrhage is complex and linked to a defect in cerebral autoregulation [2]. In severe carotid stenosis, as in our patient’s case, the chronic reduction in cerebral blood flow induces compensatory changes. This leads to the dilation of the small vessels, with a loss of their ability to modify their resistance in response to changes in pressure or flow: the principal mechanism of cerebral autoregulation. Carotid reperfusion is accompanied by hyperperfusion, leading to edema and rupture of the small vessels, in the absence of autoregulatory capacity, which can result in hemorrhage, sometimes macroscopic [5,6]. This hemorrhage is favored by endothelial lesions secondary to atherosclerosis [7]. Rupture of small vessels occluded by distal emboli originating at the surgical site, and a possible cause of cerebral hemorrhage post-coronary reperfusion. Some studies [8,9] have suggested that intracerebral hemorrhage involves the perforating arterioles of the basal ganglia in the same way as in arterial hypertension. Risk factors have been identified, including severe carotid occlusion, contralateral occlusion, poor collateral network, perioperative hypertension, recent cerebral ischemia, and excessive heparin use [7,10]. Diagnostic management relies on brain imaging to identify the hemorrhage. In our case, a cerebral ultrasound showed an image compatible with an intracerebral hematoma: a hyperechoic image within the cerebral parenchyma, leading to suspicion of the hemorrhagic origin of the deficit, which was confirmed by a CT scan. This shows the growing interest in the morphological study of the cerebral parenchyma by ultrasound, in addition to the hemodynamic study by transcranial Doppler. After confirmation, the need for neurosurgery is discussed, including evacuation of the hematoma, external ventricular bypass, or decompressive craniectomy. The rest of the management consists of controlling the bleeding by controlling blood pressure, interrupting any anticoagulant or antiaggregant therapy, or even antagonizing its effects (protamine), ensuring cerebral perfusion, and preventing secondary aggressions.

## Conclusions

Our observation reveals the dual value of cerebral Doppler ultrasonography. First, preventive, by monitoring the evolution of villi in post-reperfusion and detecting the risk of hyperperfusion hemorrhage. Second, diagnostic, allowing orientation of postoperative cerebral lesions. Cerebral complications after carotid revascularisation surgery are not only ischemic but also have a poor prognostic value. Physicians should know the risk factors and adapt their practice to avoid complications.
